# Cardiac Screening: An Important Diagnostic Tool in the Early Identification of High-Risk Children with Post-COVID-19 Multisystem Inflammatory Syndrome in Children

**DOI:** 10.21315/mjms2022.29.3.15

**Published:** 2022-06-28

**Authors:** Rameesha Shafqat, Kubra Fatima, Anisa Begum, Shafqat Qamer

**Affiliations:** 1 Dow University of Health Sciences, Karachi, Pakistan; 2 Department of Basic Medical Science, College of Medicine, Prince Sattam bin Abdul Aziz University, Alkharj, Saudi Arabia

Dear Editor,

Early studies had shown that the pediatric population was relatively spared from the effects of the coronavirus pandemic. However, the recent spike in pediatric cases of COVID-19 worldwide, including Pakistan, suggests a hyper-inflammatory condition mimicking Kawasaki disease, toxic shock-like syndrome and secondary haemophagocytic lymphohistiocytosis/macrophage activation syndrome (1) was found in a large number of pediatric patients. The surge in reported cases led it to be recognised as a distinct clinical entity named multisystem inflammatory syndrome in children (MIS-C), also often termed as a pediatric multisystem inflammatory syndrome (PMIS) (2).

MIS-C is a life-threatening infection identified by extensive inflammation, fever, enlarged lymph nodes, gastrointestinal symptoms, conjunctivitis and generalised erythema. Children manifest these signs/symptoms around 1 month following coronavirus infection (3). Cases are presented as multiorgan dysfunction and consistently elevated markers of inflammation in its severe form (4). Some patients also reported having developed neurologic manifestations, which included headache, irritability and encephalopathy (1).

A recent study by Sperotto et al. has shown that a high percentage of previously healthy children also ended up with serious cardiac complications in MIS-C (2). Likewise, further studies have revealed that initially, they have shown at least one abnormal cardiac test, which highlighted the importance of cardiac workup in assessing the severity of MIS-C and preventing future cardiac complications.

An observational cohort study conducted by Sanil et al. (5) in Children’s Hospital of Michigan showed that approximately half of the children were hypotensive, tachycardic with reduced left ventricular (LV) systolic function and manifesting signs of decreased cardiac output at the time of admission or evolved early following it.

Echocardiography plays a vital role in the diagnosis and evaluation of cardiovascular outcomes. Echocardiography reveals the main features of cardiac involvement in MIS-C, including reduced LV function, coronary artery abnormalities, dilation or aneurysm, mitral valve regurgitation and pericardial effusion (6). It also demonstrates that children with MIS-C, having depressed left ventricular function at initial presentation, are prone to high chances of acute adverse clinical course (5). Moreover, patients who had an adverse clinical course and were admitted for intensive care treatment were noted to have elevated levels of inflammatory and cardiac biomarkers (troponin, B-type natriuretic peptide (BNP), pro-B-type natriuretic peptide (proBNP) at the time of presentation (7). Another study reveals a 19% prevalence of first-degree atrioventricular block in patients diagnosed with MIS-C (8) but it is unclear whether it leads to severe outcomes in MIS-C patients. Therefore, more studies are required for this purpose.

There is a recent significant spike in coronavirus cases in children in different regions of the country. Unfortunately, in our setup, there are limited resources and facilities. There is an immense possibility that pediatric patients diagnosed with mild to moderate MIS-C, not showing features of cardiac involvement, are not being frequently monitored for cardiac parameters. This could lead to increased mortality in children and adolescents and may be associated with severe clinical outcomes. Therefore, it is emphasised that a basic cardiac workup should be organised for all patients recently diagnosed with MIS-C. The workup should aim at confirming cardiac involvement with a hemodynamic compromise during the treatment of MIS-C. The workup should also include therapeutic guidance on the serial echocardiographic assessment of cardiac function and monitoring of the brain natriuretic peptide and troponin levels. The workup may also focus on supportive care to maintain hemodynamic stability and ensure adequate systemic perfusion. Such proactive measures can lead to decreased ICU admissions and assist in the early identification of high-risk patients.
